# Supplementary material for: Human Gut Microbiome Response Induced by Fermented Dairy Product Intake in Healthy Volunteers
Source: Nutrients. 2019 Mar 4;11(3):547. doi: 10.3390/nu11030547 (PMC6470569; doi:10.3390/nu11030547)
Supplement: Supplementary file 1 [file nutrients-11-00547-s001.zip › nutrients-442893-Supplementary/Supplementary_Methods (2).docx]

**SUPPLEMENTARY METHODS**

## **Inclusion Criteria**

- Caucasian males and females;
- Age of 18-40 years;
- Verified diagnosis “healthy” according to the data of standard clinical, laboratory, and instrumental examination methods;
- Body weight within the range from 18–28 kg/m^2^;
- Signed informed consent form in the “Information sheet of a subject participating in a bioequivalence study”;
- For females: negative pregnancy test and consent to adhere dependent contraception methods for all period of study participation;
- Agreement to avoid consumption of other fermented milk or pre-/probiotic-fortified products during the study.

## **Non-Inclusion Criteria**

- Anamnestic data for individual intolerance (lactase failure) and/or emergence of alimentary allergy to components of the investigational product;
- Under 18 years old and older than 40 years;
- Body weight index ≤18 and ≥28;
- Chronic disease of digestive tract or cardiovascular, pulmonary, neuroendocrine systems, kidneys, blood;
- Surgical interventions on digestive tract (except appendectomy);
- Acute infectious, non-infectious, and allergic diseases within less than four weeks prior to the study initiation;
- Use of antibiotics within less than four weeks before the study initiation;
- Systolic blood pressure level measured in the sitting position lower than 120 mm Hg or higher than 140 mm Hg and/or diastolic blood pressure level lower than 80 mm Hg or higher than 90 mm Hg at each study period;
- Heart rate lower than 60 bpm at the time of screening or higher than 90 bpm at screening and before use of the study product at each study period;
- Regular use of prescription and over-the-counter drugs within less than four weeks prior to the study initiation; intake of dietary supplements, specialized dietary (prophylactic and treatment) products earlier than two weeks before the study initiation;
- Use of more than 10 U of alcohol per week (1 U of alcohol is equivalent to ½ l of beer, 200 mL of wine or 50 mL of spirits) or anamnestic data of alcoholism, addiction, drugs abuse;
- Pregnancy or breastfeeding;
- Smoking;
- Participation in the study with another specialized nutrition product or drug earlier than three months before the study initiation;
- Lack of the signed informed consent;
- Any reason which by the investigator’s opinion will impede subject study participation.

## **DNA Extraction and Sequencing**

Stool sample (150–200 mg) was placed in 2.0 mL tube containing 3:1 mix of 0.1 mm and 0.5 mm pre-sterilized glass beads (Sigma, USA). Then 1 mL of a warm 60 °С lysis buffer (500 mM NaCl, 50 mM Tris-HCl, pH 8.0, 50 mM EDTA, 4% SDS) was added. The mixture was vortexed and homogenized with MiniLys (Bertin Technologies, USA) at highest speed for three minutes. The lysate was incubated at 70 °С for 15 minutes and centrifuged for 20 minutes at 14,000 rpm. One milliliter of supernatant was transferred to sterile tube and put on ice. The pellet was added to a 1 mL of lysis buffer and the homogenization process was repeated. The supernatants were combined in 15 mL tubes with the addition of 4 mL of 96% ethanol and 200 μL of 3 M sodium acetate. The mixture was incubated at –20 °С for not less than 1 hour. The mixture was centrifuged for 15 minutes at 14000 rpm at +4 °С, supernatant was discarded, the DNA pellet was washed twice with 80% ethanol. The pellet was dried at 53 °C for 30–60 minutes and resuspended in 200 μL of sterilized milliQ water. The mixture was centrifuged and transferred into new tubes. Resulting DNA solution was treated with 10 μL of RNAse A (5 mg/mL) for one hour at 37 °С, followed by additional round of chloroform purification. Chloroform was added to the solution in 1:1 ratio, tube was vortexed for one minute and centrifuged at 5000× *g* for five minutes. Aqueous phase was transferred to new sterile tube and used for PCR dilutions. The obtained DNA solution was stored at –20 °С.

The amplification and sequencing as well as demultiplexing of a variable region V4 of 16S rRNA gene was performed as described before [1].

## **Statistical Analysis**

*Data Preprocessing*

Rare taxa (with maximum abundance across all samples less than five reads and non-zero abundance in less than 10 samples after rarefaction) were filtered out from abundance tables. After the filtering tables were renormalized.

*Analysis of the Metabolic Potential*

For each sample, the OTU relative abundance vector was processed using PICRUSt software to predict the relative abundance of the KEGG Orthology Groups gene groups. The KEGG metabolic pathways and modules for which the relative abundance changed significantly in the metagenomes of the volunteers after the FDP consumption were detected using the piano algorithm (“runGSA” function of “piano” R package) with the following parameters: gene set analysis method: “reporter features”, significance threshold: adjusted *p*-value threshold < 0.01.

*Correlation Analysis for Discovery of the Potential Cooperatives*

Putative interactions between the relative abundance values across all samples was calculated for all pairs of microbial genera using SpiecEasy algorithm. The visualization in the form of a co-occurrence network was constructed using the igraph R package (“igraph” function with layout method “layout.fruchterman.reingold”). The cooperatives were identified as the connected components of the network including not less than three vertices (only the positive correlation edges were considered here).

*Comparative Analysis of the Metagenomic Data*

To detect if there were significant changes in the overall microbial community structure as the result of the FDP consumption, a pairwise PERMANOVA test was performed (with the volunteer’s ID passed to the “strata” parameter). Taxa for which the relative abundance significantly changed (increased or decreased) as the result of the FDP consumption were identified via paired comparison using metagenomeSeq, an algorithm based on cumulative-sum scaling for normalization and zero-inflated Gaussian distribution mixture model. This method was specifically designed for statistical analysis of metagenomic data obtained using marker gene (e.g., 16S rRNA) sequencing. To reduce the “noise-like” effects, the taxa present in less than 15 samples were not included in the analysis; the analysis also only included the taxa present in at least one sample with the abundance higher than fixed threshold (1% for families, 0.1% for genera, 0.01% for species and OTUs). The multiple testing adjustment was performed using the Benjamini-Hochberg method. The associations were considered significant if the adjusted p-value was less than 0.05.

*Analysis of Responders*

According to a meta-analysis, the following microbial taxa were included into the list of potential lactose fermenters (only the taxa detected in the metagenomes were included):

- *Bifidobacterium*
- *Lactobacillus*
- *Lactococcus*
- *Streptococcus*
- *Slackia*
- *Corynebacterium*
- Unclassified Enterobacteriaceae (the respective entries in the used reference database correspond to *Escherichia/Shigella*).

References

1. Klimenko, N.S.; Tyakht, A.V.; Popenko, A.S.; Vasiliev, A.S.; Altukhov, I.A.; Ischenko, D.S. et al. Microbiome Responses to an Uncontrolled Short-Term Diet Intervention in the Frame of the Citizen Science Project 2018, Nutrients, 10(5), 576.
